# Total Perineal Prostatectomy: A Retrospective Study in Six Dogs

**DOI:** 10.3390/ani12020200

**Published:** 2022-01-14

**Authors:** Daniele Zambelli, Giulia Ballotta, Simona Valentini, Marco Cunto

**Affiliations:** Department of Veterinary Medical Sciences, University of Bologna, Ozzano dell’Emilia, 40064 Bologna, Italy; daniele.zambelli@unibo.it (D.Z.); giulia.ballotta2@unibo.it (G.B.); marco.cunto@unibo.it (M.C.)

**Keywords:** perineal prostatectomy, total prostatectomy, perineal hernia, dog

## Abstract

**Simple Summary:**

Prostatectomy is a surgical procedure that involves the removal of the prostate, either partially (partial prostatectomy) or completely (total prostatectomy). Total prostatectomy is considered technically difficult to perform, carrying with it many complications and unwanted side effects. The standard total prostatectomy provides a caudal celiotomy: a pubic symphysiotomy or pubic and ischial osteotomies may be required in order to improve access to the prostate gland and the pelvic urethra. Perineal hernia refers to the herniation of pelvic and abdominal viscera into the subcutaneous perineal region through a pelvic diaphragm weakness. A concomitant prostatic disease is observed in 25–59% of cases: the prostate can remain in the physiological location or displace within the hernial contents. Surgery is the treatment of choice in case of perineal hernia. The aim of this article is to describe retrospectively the total perineal prostatectomy in dogs presenting perineal hernia with concomitant prostatic diseases which required the removal of the gland. The experience in six patients (three dogs with the prostate within hernial contents and three dogs with intrapelvic prostate) are reported as well as advantages, disadvantages, and limitations of the surgical procedure.

**Abstract:**

Perineal hernia refers to the herniation of pelvic and abdominal viscera into the subcutaneous perineal region through a pelvic diaphragm weakness: a concomitant prostatic disease is observed in 25–59% of cases. Prostatectomy involves the removal of the prostate, either partially (partial prostatectomy) or completely (total prostatectomy). In case of complicated perineal hernia, staged procedures are recommended: celiotomy in order to perform colopexy, vasopexy, cystopexy, and/or to treat the prostatic disease, and perineal access in order to repair the perineal hernia. Very few reports relate prostatectomy using a perineal approach and, to the extent of the author’s knowledge, this technique has not been thoroughly investigated in the literature. The aim of this article is to retrospectively describe the total perineal prostatectomy in dogs presenting perineal hernia with concomitant prostatic diseases which required the removal of the gland. The experience in six dogs (three dogs with the prostate within hernial contents and three dogs with intrapelvic prostate) is reported as well as advantages, disadvantages, and limitations of the surgical procedure. In the authors’ clinical practice, total perineal prostatectomy has been a useful surgical approach to canine prostatic diseases, proven to be safe, well tolerated, and effective.

## 1. Introduction

Prostatectomy is a surgical procedure that involves the removal of the prostate, either partially (partial prostatectomy) or completely (total prostatectomy). Total prostatectomy is carried out mainly in early detected non-metastatic prostatic tumors, but it can also be used in the case of severe trauma, vascular disorders due to prostate dislocation, recurrent and/or unresponsive abscesses, or cystic formations. In the last two situations, however, other options are often described, such as partial prostatectomy, omentalization, marsupialization if needed, or the placement of a drain [1,2,3]. Total prostatectomy is considered technically difficult to perform, carrying with it many complications and unwanted side effects [4,5,6,7], even if, in the authors’ experience, complications have not been so frequently encountered [8]. The literature reports a high probability of urinary incontinence following total prostatectomy [9]. According to some authors, this complication occurs in over 90% of subjects [10], while according to others the percentage varies from 33% to 100% of treated cases [1,11]. For these reasons, it is usually preferred to treat prostate diseases through a conservative approach, although in some cases it may be necessary to remove the gland. The standard total prostatectomy provides a caudal celiotomy. Given the particular localization of the gland, a pubic symphysiotomy [12] or pubic and ischial osteotomies may be required in order to improve access to the prostate gland and the pelvic urethra [12,13].

Perineal hernia refers to herniation of pelvic and abdominal viscera into the subcutaneous perineal region through a pelvic diaphragm weakness. The condition is usually seen in older male dogs. Conditions that lead to perineal hernia are poorly understood: the cause could be multifactorial and potential risk factors include breed predisposition, rectal abnormalities, hormonal factors, prostatic enlargement, prostatic cyst, pelvic masses, myopathy of the pelvic diaphragm, increase of the abdominal pressure, and tenesmus [14]. A concomitant prostatic disease is observed in 25–59% of cases: the prostate can remain in the physiological location with only minimal pelvic involvement, move caudally to pelvic localization, or displace within the hernial contents [15,16]. Surgery is the treatment of choice in the case of perineal hernia. When a concurrent surgical prostatic disease is diagnosed (complicated perineal hernia), staged procedures are recommended: first, after the repositioning of herniated organs, colopexy, vasopexy, cystopexy, and/or treatment of the prostatic disease, if requested, may be performed during celiotomy. Then, the perineal hernia is repaired as the improved visualization facilitates the execution of the sutures, reducing the probability of damaging the neurovascular structures (caudal rectal nerves) and the external anal sphincter, thus decreasing the onset of post-operative fecal incontinence and relapse [16,17,18,19]. There are very few reports relating prostatectomy using a perineal approach [6,20] and, to the extent of the author’s knowledge, this technique has not been thoroughly investigated in literature.

The aim of this article is to describe retrospectively the total perineal prostatectomy in dogs presenting perineal hernia with concomitant prostatic diseases which required the removal of the gland. The experiences of six patients (three dogs with the prostate within hernial contents and three dogs with intrapelvic prostate) are reported as well as advantages, disadvantages, and limitations of the surgical procedure. Perineal prostatectomy proved to provide some advantages compared to the celiotomic access: less trauma for the patient, more accurate haemostasis, better visualization of the gland, less tension on the anastomosis site, decreased surgical time.

## 2. Materials and Methods

Medical records were analysed to identify dogs referred for perineal hernia with concurrent prostatic disease. Dogs were included if they had undergone a total perineal prostatectomy following pre-operative clinical examination, abdominal and/or perineal ultrasound and pelvic and/or thoracic radiographic examination.

Treatment options have been discussed with the owners and potential complications were presented to them. All procedures described were performed in the course of routine clinical activity by a licensed veterinarian surgeon experienced in prostatic surgery following a normal standard protocol approved by the owners of the dogs (informed consent). Moreover, this study was carried out in accordance with the relevant guidelines and regulations required by Italian Veterinary Clinical Practice (as reported in FNOVI-Federazione Nazionale Ordini Veterinari Italiani-Deontological Guidelines, art. 15).

Pre-surgical, intra-operative, and post-surgical clinical information were collected and analyzed for each patient.

Signalment, recent medical history, clinical signs, results of pre-operative laboratory tests and diagnostic imaging, and histologic diagnosis were evaluated. Information about the duration of the prostatectomy (from the skin incision to the apposition of the last suture of the vesicourethral anastomosis), intraoperative and postoperative complications, duration of recovery, discharge, and follow up was collected.

## 3. Surgical Technique

**Figure 1:**
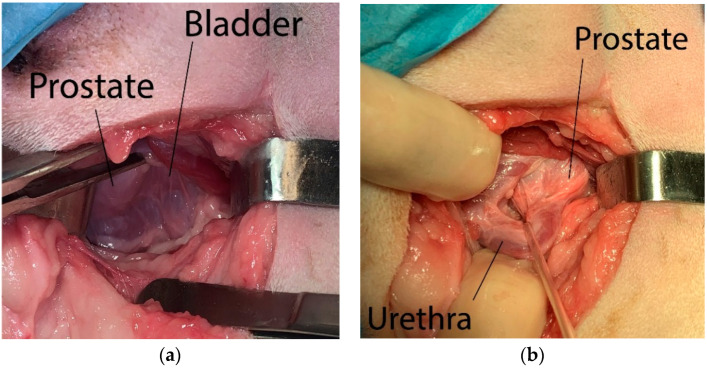
Surgery steps: (**a**) the prostate and the bladder are identified after perineal incision and blunt dissection; (**b**) a sterile disposable Penrose drain set or a silicone vascular tape was then positioned around the urethra to better identify it during the subsequent steps of the procedure.

**Figure 2:**
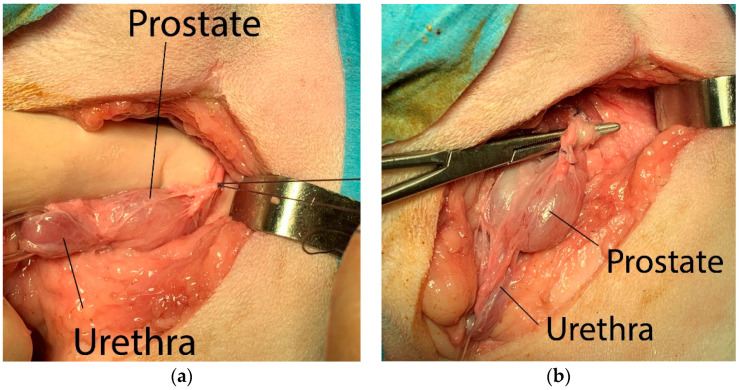
Surgery steps: (**a**) ligatures of the the neuro-vascular bundles located dorso-laterally to the prostate; (**b**) the vas deferens were identified and ligated before the gland was tractioned caudally.

**Figure 3:**
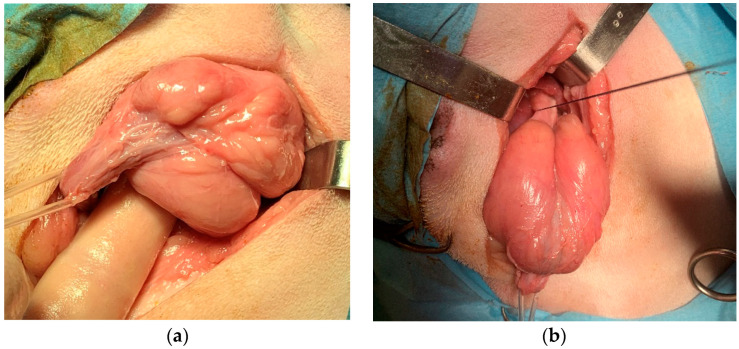
Surgery steps: (**a**) the prostate was tractioned caudally; (**b**) stay suture placement in the neck of the bladder.

**Figure 4:**
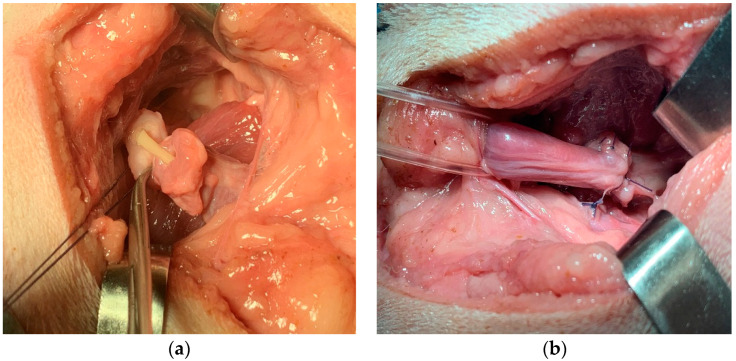
Surgery steps: (**a**) the catheter is reintroduced into the urethra to realign the stumps and allow the vesicourethral anastomosis; (**b**) vesicourethral anastomosis.

The perineal region was aseptically prepared for surgery. Fecal material was removed from the rectum and the anal sacs were evacuated. A temporary pursue string suture was placed in the anus and a sterile urinary catheter (Dog Catheter with Female Luer Mount, Portex^®^, Smiths Medical International Lt., Hythe, Kent, UK) was placed in the urethra. The dog was positioned in ventral recumbency with the hind limbs pulled caudally at the edges of the operating table and the tip of the tail was fixed over the dog’s back. A pad was placed under the caudal abdomen to determine a slight upward inclination of the hindquarters: the same result can be obtained by tilting the operating table (Trendelemburg or anti-shock position). To allow the reader a better understanding of the different surgical steps and a good visualization of the anatomical structures, Figure 1, Figure 2, Figure 3 and Figure 4 have been taken from a cadaver study.

A skin incision was made over the hernia, from the base of the tail until a midpoint between the pubis and ischial tuberosity, crossing it by at least 2–3 cm. The incision line was curved, so that in its intermediate portion it moves away from the anus by 2–4 cm. The subcutaneous tissue was detached through a blunt dissection to expose the hernial contents, which were repositioned cranially into the pelvic cavity. Starting from the root of the penis and finding the rectum dorsally, the urinary catheter previously inserted in the urethra was detected through digital palpation, allowing to identify the prostate more easily (Figure 1a). The membranous urethra was identified by palpating the urinary catheter with a finger and isolated from the surrounding tissues. A sterile disposable Penrose drain set or silicone vascular tape was then positioned around the urethra to better identify it during the subsequent steps of the procedure (Figure 1b).

The prostate was isolated from the surrounding periprostatic adipose tissues by blunt dissection, taking care to tie the neuro-vascular bundles located dorso-laterally to the gland (Figure 2a), making the ligatures as close to the gland as possible. The vas deferens were identified and ligated before the gland was tractioned caudally (Figure 2b).

The prostate was gently tractioned caudally (Figure 3a). A stay suture was placed in the neck of the bladder to ensure that it could always be identified, did not retract into the abdomen, and did not undergo twisting before the anastomosis (Figure 3b). The urethra was transected just proximally and distally to the prostate capsule, progressively withdrawing the urinary catheter into the membranous urethra. The prostate was then removed. The catheter was advanced again into the bladder (Figure 4a). The end-to-end vesicourethral anastomosis was carried out with single interrupted absorbable monofilament 4-0 sutures (Figure 4b).

All the sutures were always applied in such a way that they are diametrically opposite, in order to guarantee the symmetry of the anastomosis. Care should be taken to not involve the mucosal layer and catheter in the suture. At the end of the anastomosis, the stay suture previously applied to the bladder neck was removed.

The bladder was repositioned in the abdomen and the simple male urethral catheter was replaced with a Foley catheter (Well Lead Medical Co., Ltd., Guangzhou China). The hernia was then repaired according to the technique selected by the surgeon, namely traditional herniorrhaphy or prosthetic implant. An orchiectomy was performed if the dog was intact.

In the case of a prostate completely displaced in the hernial contents, the identification of the prostate occurs very easily when the hernial sac is opened. The subsequent steps are similar to those described above.

## 4. Results

Six client-owned dogs (four intact males and two neutered males) of different breeds (four mixed-breed dogs, one Maltese and one Lagotto Romagnolo), mean age 8.33 ± 1.80 years (median age eight years; range 6–11 years) and mean weight 13.55 ± 5.28 kg (median weight 15 kg; range 5.2–20 kg) were included in the study. A perineal hernia with concomitant prostatic disease was diagnosed in all dogs and the removal of the prostate was recommended.

The clinical signs manifested on presentation were: swelling of the perineal region (six in six), lethargy (five in six), fecal tenesmus (five in six), anorexia (three in six), hypothermia (two in six), tachycardia (two in six), mucosal congestion (two in six), abdominal pain (two in six), gait abnormality (two in six), macroscopic haematuria (one in six), intermittent urethrorrhagia (one in six), seizures (one in six).

Preoperative serum biochemistry and haematology test were performed in all dog as well as urinanalysis.

Dogs underwent abdominal ultrasound (six in six), ultrasound of the perineal region (six in six), radiographic examination of the abdominal-pelvic region (six in six), thoracic radiography (one in six) and positive-contrast retrograde cystourethrography (two in six).

**Figure 5:**
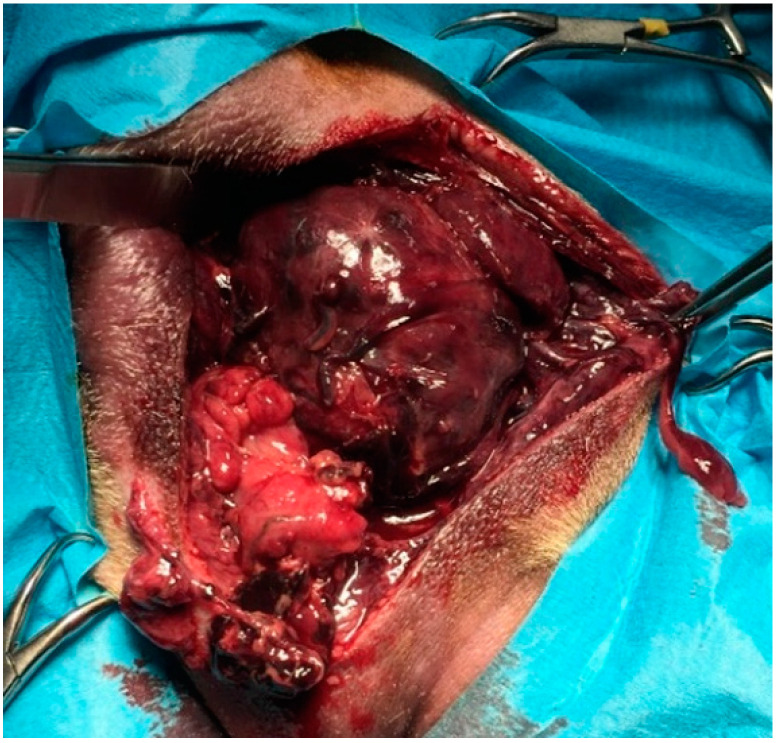
8-year-old mixed breed dog. Prostate displacement in the hernial contents: macroscopic appearance of the gland.

The diagnosis was unilateral right perineal hernia (three in six), bilateral perineal hernia (three in six): in three dogs the prostate was totally displaced in the hernial contents, whereas in three dogs a partially dislocated or intrapelvic prostate was observed. Concomitant prostatic diseases detected were prostatitis and prostatic abscesses (three in six), benign prostatic hyperplasia with severe large cysts with urinary content (one in six), prostatitis and severe parenchymal necrosis (one in six) (Figure 5), prostate adenocarcinoma (one in six). Clinical findings, laboratory tests and diagnostic imaging oriented the surgeon towards the decision to remove the prostate. Preoperative findings, including cytologic samples obtained by US-FNA and/or prostatic massage, agreed with final histologic diagnoses in all cases.

Signalment, hernia, and prostatic localization details, clinical findings, preoperative haematology and biochemistry tests, urinalysis results, radiographic and echographic results, and histologic diagnosis for dogs who had undergone perineal prostatectomy are summarized in Table 1 and Table 2.

The operative time, defined as the time between the skin incision and the last suture of the vesicourethral anastomosis, was 44.17 ± 1.86 min (median 45 min). Duration of hospitalization was 4.83 ± 2.19 days (median six days). No intraoperative complications, such as intraoperative haemorrage or urinary leakage from the anatostomotic site, were observed in any dog. Dogs were ambulatory 12 h after surgery.

In the hospitalization period, dogs were provided with antibiotic therapy (marbofloxacin 3 mg/kg SID) and analgesics (buprenorfine 15 mcg/kg TID).

Four dogs survived after the discharge from the hospital, while one dog (Table 1; case 1) died at the end of surgery due to unresponsive cardiorespiratory failure (his conditions were already critical at the time of admission to the hospital) and one dog (Table 1; case 3) was euthanized after a week for progressive clinical and laboratory worsening, above all renal values. Among the rest of the dogs, one dog (Table 1; case 6) died after seven months for local tumour recurrence; the remaining three dogs (Table 1; cases 2, 4, 5) were monitored with telephone follow-ups for six months from the discharge.

No postoperative complications were reported during hospitalization in survivors (four in six), except a 10-year-old mixed breed (case 5), which, after the urinary catheter removal, manifested fecal tenesmus and intermittent urinary incontinence only during walking, returning to complete urinary continence within a few weeks, as reported by the owners. In the remaining animals, owners reported the dogs were fully continent and micturition was normal throughout the follow-up period or, in case 6, up to the time of death.

## 5. Discussion

Perineal hernia is defined as the dislocation of viscera and/or organs due to a pelvic diaphragm weakness: different structures, such as prostate, bladder, and portions of the intestine, can be involved. In the case of concomitant prostate disease, a conservative approach is usually preferred, considering that prostatectomy is an intervention that can lead to the complications described above. In some cases, however, it is necessary to remove the gland. If the prostate is included in the hernial contents and it is necessary to proceed with a total or partial prostatectomy, a two-step approach is usually performed: celiotomy to reposition the organ in place and remove it, perineal approach to repair the hernia. The literature reports cases of involuntary perineal prostatectomy in the case of surgical treatments aimed at resolving perineal hernias whose contents also consisted of prostate and urethra [21,22], or in cryptorchidism surgery [23,24,25]. The cases described referred to dogs whose prostate had been removed by mistake in a previous surgery, requiring subsequent corrective interventions. Some authors cite the possibility of using the perineal approach to remove the prostate [6,20]. To the best of the writer’s knowledge, it does not appear that the perineal approach has been thoroughly described yet.

The aim of this article is to describe the perineal prostatectomy performed on six male dogs referred for perineal hernia with concomitant prostatic diseases which required the removal of the gland and to evaluate any complications and outcomes, comparing the technique with the standard total prostatectomy via celiotomy. However, the comparison between our data and those present in the literature has been very complicated as relevant studies described total prostatectomy by celiotomy without reporting details such as duration of the surgery or intraoperative complications [1,6,7,11,26,27]. The indication for performing perineal approach in the dog is a perineal hernia with concurrent prostatic disease requiring the removal of the gland.

Four intact males and two castrated males of different age and breed were examined, confirming the literature regarding the lack of a particular breed predisposition and the average age at which the problem that required the prostatectomy arose. The common condition of all the dogs in our study was a perineal swelling: the symptoms reported are similar to those reported in the literature [11,28,29]. As for the changes detected by the blood biochemical tests, there was a great variability between subjects, probably due to the fact that our sample included dogs presenting different prostatic diseases and clinical findings. Urinalysis showed hematuria in all dogs and bacteriuria in two dogs, similarly to what was described by Bennett et al. Prostatomegaly was observed in all dogs: the size of the prostatic gland and its position were defined by transrectal palpation and imaging, in order to choose the surgical approach. In all cases in which the prostate was displaced in the hernial contents, the macroscopically detectable alterations of the prostate (Figure 5) were a primary indication for a perineal prostatectomy. In cases in which the prostate was intrapelvic, the condition of prostate, periprostatic tissues, and the patients’ current situation favored prostatectomy over other therapeutic techniques or pharmacological therapies. However, it was mandatory to evaluate the size with respect to the pelvic cavity, comparing the size of the prostate with the distance between the floor of the pelvis and the sacral vertebral bodies in a radiographic lateral view.

The diagnosis of infected cysts located in the perineal hernia was decisive for choosing the perineal prostatectomy, since the risk of breaking these cysts by manipulating them was greater by celiotomy. Unlike what is reported in the bibliography [11], no CT or MRI was used as the owners did not consent to the execution of these procedures, but we believe that these exams could provide very precise information concerning prostate enlargement.

All dogs underwent total perineal prostatectomy with a 45 min average duration of surgery, defined as the interval from the skin incision to the apposition of the last suture point of the vesicourethral anastomosis. Unfortunately, these data do not find a possibility of comparison, as the average duration of standard total prostatectomy interventions is not reported in the literature [11,30]. However, it should be emphasized that unpublished data of the authors attest to the average time required for a standard procedure being around 70–90 min.

Two dogs died from causes not related to prostatectomy, one dog died after seven months due to the recurrence of the tumor, while the other three are still alive. Vlasin et al. report the euthanasia of most of the dogs undergoing total removal of the prostate by celiotomy: some subjects were euthanized five, 10, and 14 days after surgery because of serious complications, such as cardio-circulatory arrest, haemorrhage, shock, acute renal failure, urinary leakage through the anastomotic site, and perforation or stricture of the urethra. In the study by Bennett et al., the overall median survival time after prostatectomy was 231 days [11]. It should be noted that Bennett et al. and Vlasin et al. referred to cancer patients, in which survival was reduced due to the presence of metastases or tumor recurrence [11,30]. Zambelli et al. reported a survival of 4–9 months after standard total prostatectomy in the case of prostatic neoplasms. The authors described no complications and/or urinary incontinence, while they highlighted the improvement in the expectation and quality of life of patients [8].

Total prostatectomy is a procedure not routinely performed due to the high risk of intra- and post-operative complications. In our study, we only recorded a minor complication related to the removal of the urinary catheter by one dog in the hospitalization period.

Urinary incontinence is reported as a possible major complication and could undergo spontaneous resolution in the weeks following the prostatectomy [1,9]. None of our dogs was incontinent prior to surgery and this has been a very important part of the decision process, as incontinence and its impact on quality of life must be carefully discussed with the owner. Patients had no symptoms of urinary incontinence after perineal prostatectomy except one dog, who presented with fecal tenesmus and intermittent urinary incontinence during walking in the four days following discharge: the owner reported that symptoms gradually resolved within a few weeks. Other complications described but not detected in this study are fecal incontinence, polyuria/polydipsia, recurrence of symptoms (dyschezia, dysuria, haematuria), delayed healing or wound infection [30], urinary infections, wound dehiscence, uroabdomen, and prepubic hernia [11].

The data obtained from clinical experience, even if derived from a numerically limited sample, indicate that total perineal prostatectomy has some intraoperative advantages over celiotomy in the case of perineal hernia concomitant with a prostatic disease requiring gland removal. Perineal prostatectomy allows to perform a single operative access and to avoid changing the animal’s recumbency during the surgery, contributing to maintain the sterility of the operating field.

Clinical experience allows us to state that total perineal prostatectomy required less time than the standard prostatectomy, which involves repositioning the dislocated prostate and its removal after celiotomy, or directly a celiotomy approach if the gland is not dislocated, both procedures associated with the reduction of the perineal hernia. The operative time decrease also affects the duration of the anaesthetic procedure and the amount of drugs administered to the patient.

A further advantage was the improved visualization and mobilization of the prostate, avoiding pelvic osteotomy as necessary during a celiotomic prostatectomy, and reducing the invasiveness and duration of the procedure, postoperative pain, hospitalisation period, and limitation of the patient’s physical activity. The prostate vessels could be better visualized, and their ligation carried out more simply. Therefore, intra-operative haemostasis was more easily performed. The prostatic neuro-vascular bundles located on the dorsal surface of the gland can be legated or coagulated: the excellent visualization of the operative field due to the perineal approach allowed us to use standard surgical techniques and to ligate the vessels without lengthening the operating times. No intraoperative heamorrage was observed in any patient. We did not coagulate the vessels, but it is presumable that the use of clamping devices such as Ligasure could further reduce the length of surgery.

A particularly delicate aspect of prostatectomy is related to vesicourethral anastomosis which, given the very caudal position of the urethra, is considered one of the most complex aspects of a prostatectomy. In the case of total perineal prostatectomy, the urethral stumps to be anastomosed are more visible and easier to affix and suture than in standard prostatectomy.

Finally, if there were abscesses or cystic formations involving the prostatic parenchyma or heading more caudally, the risk of cyst rupture and following contamination of the operating field were decreased, unlike what could happen in the celiotomy approach.

Therefore, in our experience, we have not highlighted any particular drawbacks related to the technique. However, this approach does not allow to create surgical adhesions, which in some cases of perineal hernia correction are considered necessary (for example, colopexy).

The only contraindication related to the surgical technique in the case of an intrapelvic pathological prostate is linked to the size of the gland: if the gland is so enlarged as to not allow traction towards the perineal region, a perineal access is not recommended. Even if prostatomegaly is accompanied by dislocation of the prostate towards the cranio-ventral portions of the abdomen, it is not recommended to carry out the perineal approach. It is therefore mandative to perform accurate pre-operative diagnostic imaging to identify the location and size of the gland, in order to choose the most suitable surgical approach.

The main limitation of our study could be represented by the small number of patients undergoing total prostatectomy. However, it is a fact that, except for case reports [5], the literature reports very small sample sizes: the largest treatment group is the one described in 2018 by Bennet et al., who carried out a retrospective multi-institutional case series study [11], followed by Vlasin et al., who carried out a prospective clinical trial on total prostatectomy in 10 dogs [30].

The last limitation is inherent to retrospective studies: medical records can be incomplete and there could be a lack of standardization of main data collection. However, in our study, the standardization of intra operative treatment has always been guaranteed, as the surgeon performing the procedure—a surgeon experienced in prostatic surgery—did not differ in all cases.

## 6. Conclusions

Total perineal prostatectomy can represent a useful surgical approach to prostatic diseases and in our experience has proven to be safe, well tolerated, and effective in dogs affected by perineal hernia. Conservative therapy is always preferable, but when there are no alternatives or there are indications for the prostatectomy, perineal access is a surgical approach that makes the removal of the prostate easier and faster than a celiotomic one. The intra- and peri-operative results have been rather encouraging. However, additional cases will be necessary to refine this technique and peri-surgical protocol, prompting us to hypothesize in the future a possible use of this approach for prostatic diseases even in the absence of contemporary perineal hernia.

## Figures and Tables

**Table 1:** Signalment, hernia and prostatic localization details, clinical findings for dogs undergone perineal prostatectomy.

Case	Signalment	Perineal Hernia	Clinical Findings
1	8 yo mixed breed	Right unilateral Prostate within the hernial contents	Swelling of the perineal region, lethargy, anorexia, hypothermia, tachycardia, mucosal congestion, seizures
2	9 yo mixed breed	Right unilateral Intrapelvic prostate	Swelling of the perineal region, lethargy, fecal tenesmus, anorexia, abdominal pain, haematuria, gait abnormalities
3	11 yo Maltese	Bilateral Prostate within the hernial contents	Swelling of the perineal region, lethargy, fecal tenesmus, hypothermia, tachycardia, mucosal congestion
4	7 yo Lagotto Italiano	Right unilateral Partially intrapelvic prostate and prostatic cyst within the hernial contents	Swelling of the perineal region, fecal tenesmus, abdominal pain, intermittent urethrorrhagia
5	10 yo mixed breed	Bilateral Prostate within the hernial contents	Swelling of the perineal region, lethargy, fecal tenesmus, stranguria
6	6 yo mixed breed	Bilateral Intrapelvic prostate	Swelling of the perineal region, lethargy, fecal tenesmus, anorexia, gait abnormalities

**Table 2:** Preoperative hematology and biochemistry tests, urinalysis results, radiographic (RX) and echographic (US) results, final diagnosis for dogs undergone perineal prostatectomy; BPH = benign prostatic hyperplasia.

Case	Hematology and Biochemistry	Urinalysis	RX	US	Diagnosis
1	AST 331 U/L (20–42); ALP 533 U/L (42–180) Creatinine 2.79 mg/dL (0.65–1.35) Urea 150.36 (18–55); GGT 20.1 U/L (0–5.8) PT 9.5 s (5.0–7.5); aPTT 17.6 s (8.0–16.5)	Hematuria Leucocytes Proteinuria *Staphylococcus* spp.	Pelvic region	Prostate 3.5 × 4.4 cm Irregular pattern	Prostatitis with disseminated necrotic areas
2	Hgb 24.3% (12–18); HCT 69.8% (37–55) RBC 10,600,000 (5,500,000–8,500,000) WBC 20,360/mm^3^ (6000–17,000) Albumin 2.13 g/dL (2.75–3.85)Albumin/globuline 0.41 mg/dL (0.75–1.35) Urea 64.03 mg/dL (17–48)	Hematuria Leucocytes	Pelvic region	Prostate 4.60 × 4.71 cm Anechoic intraparenchymal area (2.80 × 3.24 cm) Suspected prostatic abscess	Prostatitis and prostatic abscess
3	Creatinina 1.61 mg/dL (0.75–1.4) Urea 81 mg/dL (17–48); GGT 9.5 U/L (0–5)	Hematuria Leucocytes ProteinuriaRod-shaped bacteria	Abdominal and pelvic regions Positive-contrast retrograde cystourethrography	Prostate 2.6 × 3.6 cm Anechoic intraparenchymal area (1.5 × 2.6 cm) Suspected prostatic abscess	Chronic prostatitisand large prostatic abscess
4	MPV 18.3 fL (6.6–10.9); AST 58 U/L (15–52) ALT 73 U/L (15–65)	Hematuria	Abdominal and pelvic regions	Prostate 4.55 × 4.62 cm Anechoic intraparenchymal area (3.50 × 3.10 cm)	BPH and prostatic cyst
5	WBC 26,640/mm^3^ (6000–17,000) Urea 199.75 mg/dL (17–48) Creatinine 7.65 mg/dL (0.75–1.4)	Hematuria Leucocytes Proteinuria	Abdominal and pelvic regions Positive-contrast retrograde cystourethrography	Prostatic size were not ultrasonographically investigated. Two anechoic area occupied almost completely the gland. Suspected prostatic abscess	Prostatitis and large prostatic abscesses involving the whole gland
6	Neutrophils 13,188/mm^3^ (3000–12,000) Eosinophils 785/mm^3^ (0–750) ALT 122 U/L (12–65)	Hematuria Leucocytes	Abdominal and thoracic	Prostate 3.22 × 3.87 cm, Irregular pattern and mineralization	Adenocarcinoma

## Data Availability

All data are contained in the manuscript.
